# Association between type of intervention center and outcomes after endovascular treatment for acute ischemic stroke: Results from the MR CLEAN Registry

**DOI:** 10.1177/23969873221145771

**Published:** 2022-12-22

**Authors:** Susanne GH Olthuis, Wouter H Hinsenveld, Florentina ME Pinckaers, Marzyeh Amini, Hester F Lingsma, Julie Staals, Tobien HCML Schreuder, Wouter J Schonewille, Lonneke SF Yo, Yvo BWEM Roos, Alida A Postma, Diederik WJ Dippel, Wim H van Zwam, Robert J van Oostenbrugge, Inger R de Ridder

**Affiliations:** 1 Department of Neurology, Maastricht University Medical Centre and School for Cardiovascular Diseases (CARIM), Maastricht, The Netherlands; 2 Department of Radiology and Nuclear Medicine, Maastricht University Medical Centre and School for Cardiovascular Diseases (CARIM), Maastricht, The Netherlands; 3 Department of Public Health, Erasmus MC, University Medical Centre, Rotterdam, The Netherlands; 4 Department of Neurology, Zuyderland MC, Heerlen, The Netherlands; 5 Department of Neurology, Sint Antonius Hospital, Nieuwegein, The Netherlands; 6 Department of Radiology, Catharina Hospital, Eindhoven, The Netherlands; 7 Department of Neurology, Amsterdam UMC, Location University of Amsterdam, Amsterdam, The Netherlands; 8 Department of Radiology and Nuclear Medicine, Maastricht University Medical Centre and School for Mental Health and Sciences (MheNS), Maastricht, The Netherlands; 9 Department of Neurology, Erasmus MC, University Medical Centre, Rotterdam, The Netherlands

**Keywords:** Acute ischemic stroke, endovascular treatment, center type

## Abstract

**Background:**

Endovascular treatment (EVT) for acute ischemic stroke (AIS) is performed in intervention centers that provide the full range of neuro(endo)vascular care (level 1) and centers that only perform EVT for AIS (level 2). We compared outcomes between these center types and assessed whether differences in outcomes could be explained by center volume (CV).

**Patients and methods:**

We analyzed patients included in the MR CLEAN Registry (2014–2018), a registry of all EVT-treated patients in the Netherlands. Our primary outcome was the shift on the modified Rankin scale (mRS) after 90 days (ordinal regression). Secondary outcomes were the NIHSS 24–48 h post-EVT, door-to-groin time (DTGT), procedure time (linear regression), and recanalization (binary logistic regression). We compared outcomes between level 1 and 2 centers using multilevel regression models, with center as random intercept. We adjusted for relevant baseline factors, and in case of observed differences, we additionally adjusted for CV.

**Results:**

Of the 5144 patients 62% were treated in level 1 centers. We observed no significant differences between center types in mRS (adjusted(a)cOR: 0.79, 95% CI: 0.40 to 1.54), NIHSS (aβ: 0.31, 95% CI: −0.52 to 1.14), procedure duration (aβ: 0.88, 95% CI: −5.21 to 6.97), or DTGT (aβ: 4.24, 95% CI: −7.09 to 15.57). The probability for recanalization was higher in level 1 centers compared to level 2 centers (aOR 1.60, 95% CI: 1.10 to 2.33), and this difference probably depended on CV.

**Conclusions:**

We found no significant differences, that were independent of CV, in the outcomes of EVT for AIS between level 1 and level 2 intervention centers.

## Introduction

After endovascular treatment (EVT) became the standard of care for treating large vessel occlusion in patients with acute ischemic stroke (AIS), it has been internationally debated which requirements centers should meet to perform these procedures. In this context, an international consensus group with delegates from different neuro-interventional, -surgical, and -radiological associations defined three types of intervention centers.^1^ Level 1 centers provide a full range of neuro(endo)vascular care, whereas level 2 centers perform EVT for AIS but no other types of neuro(endo)vascular procedures. Level 3 stroke centers have a stroke unit and offer intravenous thrombolysis but do not perform any neuro(endo)vascular procedures. For EVT, a level 3 center will transfer eligible patients to a nearby level 1 or level 2 center.^1^

The consensus group recommended EVT for AIS to be performed in level 1 centers, except in case transfer times exceed 2 h. This recommendation was based on the assumption that level 1 centers treat higher volumes of patients than level 2 centers, which is not necessarily true. On the one hand, experience with other neuro-endovascular procedures in level 1 centers may lead to better results in performing EVT for AIS. On the other hand, treatment in level 2 centers may have a benefit over treatment in level 1 centers, due to shorter travel distances and perhaps a faster workflow.

Even though the effects of center volume and treatment delays on outcomes after EVT have been previously studied – to our knowledge – there has not yet been a comparison based on the type of intervention center (level 1 vs level 2 centers).^2,3^

In the present study, we, therefore compared clinical, imaging, and workflow outcomes between level 1 and level 2 centers, and assessed whether observed differences were independent of the annual center volume of patients treated with EVT.

## Methods

### Study design and participants

We analyzed data from patients included in the MR CLEAN Registry (a multicenter, national, prospective, observational monitoring study) between March 2014 and December 2018. The MR CLEAN Registry enrolled all patients who received arterial puncture to undergo EVT for AIS in the Netherlands. The study design and methods have been described previously.^4^

We selected patients at least 18 years of age, with a symptom onset to arterial puncture time ⩽6.5 h, and AIS due to a proximal intracranial vessel occlusion of the anterior circulation (i.e. internal carotid artery (ICA), internal carotid artery terminus (ICA-T), middle (M1/M2) cerebral artery, or anterior (A1/A2) cerebral artery) as confirmed by imaging (i.e. computed tomography angiography (CTA)).

### Outcomes

Our primary outcome was the functional outcome of patients after 90 days assessed as the score on the full distribution of the modified Rankin Scale (mRS).^5^

Secondary outcomes were: National Institutes of Health Stroke Scale (NIHSS) score at 24–48 h post-EVT; mortality at 90 days; occurrence of stroke progression (defined as an increase of at least four NIHSS score points); occurrence of new ischemic stroke; and occurrence of symptomatic intracranial hemorrhage (defined as an intracranial hemorrhage on follow-up imaging classified according to the Heidelberg criteria, which was related to an increase of at least four NIHSS points or death); successful recanalization (defined as an extended thrombolysis in cerebral infarction score (eTICI) ⩾*2B*)^6^; any procedural complications scored on digital substraction angiography (i.e. perforation, dissection, intracranial hemorrhage during the procedure, a distal thrombus, embolus in a new vascular territory, and vasospasms); achievement of first-pass reperfusion (defined as an eTICI⩾2C in the first attempt); duration of the procedure (defined as the time from groin puncture until recanalization or the last contrast bolus); and door to groin time (DTGT) (defined as the duration in minutes from arrival at the emergency room (ER) of the intervention center up until groin puncture).

### Missing data

Part of the missing NIHSS scores could be retrospectively assessed using medical records. In case of missing NIHSS scores due to the death of a patient before the assessment at 24–48 h post-EVT, we assigned the maximum score of 42 points. The number of missing values per variable is shown in the tables.

All missing values were imputed with multiple imputations by chained equations with the *mice* package, in R statistical software version 4.0.2 (R Foundation for Statistical Computation, Vienna, Austria), using relevant covariates and outcome variables present in the MR CLEAN Registry dataset.^4^

### Statistical analysis

We used crude data for the reporting of baseline characteristics and the description of our outcome variables.

Regression analyses were applied to estimate the effect of the association between center type (level 1 vs level 2 centers) and our outcome variables. In our models, the NIHSS and Alberta Stroke Program Early CT scores (ASPECTS) were used as continuous variables. Due to our sample size, there was no need to log transform NIHSS scores with a non-Gaussian distribution. We applied linear, binary, and ordinal regression models, depending on the outcome variable, and presented the results as beta coefficients (β), odds ratios (OR), or common odds ratios (cOR) respectively. We estimated the unadjusted and adjusted associations with 95% confidence interval (95% CI). In the regression analysis, mRS scores were inverted so cOR above 1.0 indicate better outcomes.

To account for data clustering, we used generalized linear mixed-effects models with center as the random intercept. We adjusted for age; sex; NIHSS score at baseline; pre-stroke mRS score; patient medical history (i.e. diabetes mellitus, atrial fibrillation, hypertension, and previous stroke); baseline blood glucose concentration; administration of intravenous thrombolysis; duration between onset to ER arrival at the intervention center; collateral grade; occlusion segment; and ASPECTS at baseline. These confounders were chosen based on their expected relationship to the outcome and previous literature.^7^

If a significant association between center level and one of our outcome variables in the adjusted model was found, we assessed whether this association was independent of center volume (CV) by adding this variable to the model. CV was defined as the number of patients treated with EVT in the center in the previous year (calculated per current procedure/patient).

The variables CV and interventionist experience might overlap, though a structural difference could exist between center types and the number of interventionists and their individual experience. Data on interventionist experience is only available for EVT-treated patients in MR CLEAN centers and not for all patients receiving groin puncture to undergo EVT (*N* = 4091). We will therefore perform a sensitivity analysis on this subset by correcting our primary analysis and the significant secondary outcomes with the number of procedures in the last year of the most experienced interventionist performing the procedure (EXPfreq).

All analyses were performed with R statistical software version 4.0.2, using the *lmer* and *glmer* functions from the *lme4* package, and the *clmm* function from the *ordinal* package.

## Results

### Participants and intervention centers

**Figure 1.**
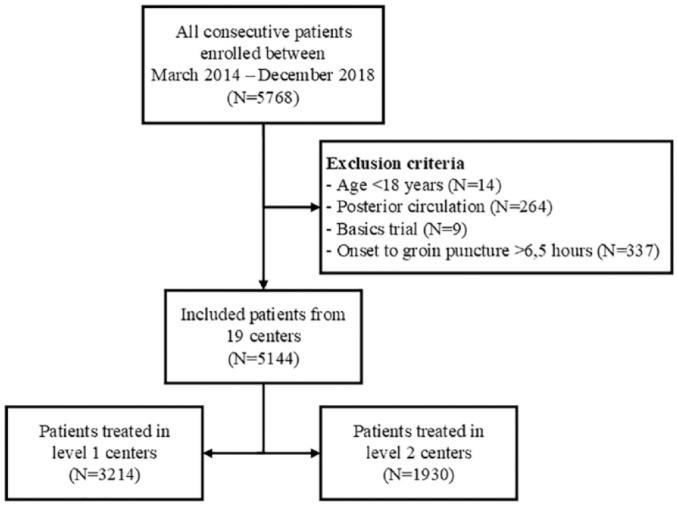
Flowchart of included patients.

The MR CLEAN Registry registered a total of 5768 patients, of whom 5144 patients fulfilled the inclusion criteria of the current analyses. There were ten level 1 centers and nine level two centers. Sixty-two percent (*n* = 3214) of the patients were treated in level 1 centers (Figure 1).

### Baseline characteristics

Patients treated in a level 1 center were on average younger; suffered less often from a medical history of hypertension, hypercholesteremia, or peripheral artery disease; had better collateral scores on baseline CTA; and arrived about 14 min later at the ER, compared to patients treated in a level 2 center (Table 1). Level 1 centers treated more referred patients and had higher patient volumes compared to level 2 centers (Table 1 and Figure S1).

**Table 1.** Baseline characteristics of patients treated in either level 1 or level 2 centers.

	Level 1 center (*n* = 3214)	Level 2 center (*n* = 1930)
Clinical characteristics		
Age–median (IQR)	72 (61–80)	74 (63–81)
Male sex	1676 (52%)	1009 (52%)
NIHSS–median (IQR)	15 (10–19), 3178	16 (11–20), 1728
Medical History		
Diabetes mellitus	530/3201 (17%)	306/1747 (18%)
Hypertension	1574/3159 (50%)	1024/1733 (59%)
Hypercholesterolemia	848/3128 (27%)	641/1680 (38%)
Atrial fibrillation	722/3187 (23%)	460/1726 (27%)
Myocardial infarction	439/3168 (14%)	269/1720 (16%)
Peripheral arterial disease	229/3182 (7.2%)	213/1704 (13%)
Previous ischemic stroke	548/3198 (17%)	339/1740 (19%)
Pre-stroke mRS score >2	418/3098 (13%)	218/1750 (12%)
Medication and intoxications		
Antiplatelet	1006/3178 (32%)	536/1737 (31%)
DOAC	157/3182 (4.9%)	82/1738 (4.7%)
Vitamin K antagonists	385/3189 (12%)	249/1748 (14%)
Smoking	672/2429 (28%)	347/1295 (27%)
Imaging characteristics		
Level of occlusion		
ICA	161/3072 (5.2%)	86/1699 (5.1%)
ICA-T	599/3072 (19%)	326/1699 (19%)
M1	1739/3072 (57%)	948/1699 (56%)
M2	550/3072 (18%)	334/1699 (20%)
Other*	23/3072 (0.7%)	5.0/1699 (0.3%)
ASPECTS–median (IQR)	9 (8–10), 3108	9 (8–10), 1708
Collaterals grade 2–3	1818/3036 (60%)	908/1665 (55%)
Workflow characteristics		
Onset to arrival ER^†^(min)–median (IQR)	132 (61–186), 3072	118 (52–175), 1809
Transfer from Level 3 center	1811/3211 (56%)	883/1865 (47%)
Off-hours^‡^	2081 (65%)	1194 (62%)
Treatment with IVT	2353/3207 (73%)	1328/1842 (72%)
Center volume^§^–median (IQR)	86 (57–127), 2765	58 (42–86), 1614

NIHSS: National Institutes of Health Stroke Scale; mRS: modified Rankin Scale; DOAC: direct oral anticoagulant; ICA: internal carotid artery; ICA-T: ICA terminus; M1/M2/M3: middle cerebral artery; A1/A2: anterior cerebral artery; ASPECTS: Alberta Stroke Program Early CT score; ER: emergency room; IVT: intravenous thrombolysis.

In case of missing values, the numbers in this table are noted as “no./total no.,” or “median (IQR), total no.”

* M3/A1/A2 occlusion.

† Arrival ER in the intervention center.

‡ Presentation Monday to Friday between 17:00 and 08:00 hours, weekends (Friday 17:00 to Monday 8:00), and national holidays.

§ Number of EVT-treated patients in the previous year (calculated per procedure).

### Clinical, imaging, and workflow outcomes

We found no significant difference between the 90-day mRS score of patients treated in level 1 centers compared to level 2 centers (adjusted(a)cOR 0.79, 95% CI: 0.40–1.54) (Table 2).

**Table 2.** Descriptives and effect estimates for the association between outcomes and treatment in level 1 versus level 2 centers.

	Level 1 center (*n* = 3214)	Level 2 center (*n* = 1930)	EE	Unadjusted^§^ (95% CI)	Adjusted^§^ (95% CI)
Clinical outcomes					
mRS score at 90 days–median (IQR)	3 (2–6), 2882	3 (1–6), 1712	cOR	0.87 (0.73 to 1.04)	0.79 (0.40 to 1.54)
NIHSS score at 24–48 h–median (IQR)	10 (4–17), 2998	9 (3–18), 1630	β	0.00 (−0.92 to 0.93)	0.31 (−0.52 to 1.14)
Mortality at 90 days	808/2882 (28%)	488/1712 (29%)	OR	0.99 (0.85 to 1.15)	1.13 (0.91 to 1.41)
*Safety outcomes*					
Stroke progression	287 (8.9%)	155 (8.0%)	OR	1.12 (0.77 to 1.61)	1.09 (0.74 to 1.59)
New ischemic stroke	53 (1.6%)	22 (1.1%)	OR	1.44 (0.68 to 3.04)	1.43 (0.70 to 2.91)
Symptomatic ICH	165 (5.1%)	125 (6.5%)	OR	0.79 (0.59 to 1.06)	0.80 (0.59 to 1.08)
Imaging outcomes					
Recanalization*	2119/3109 (68%)	945/1661 (57%)	OR	1.59 (1.11 to 2.27)	1.60 (1.10 to 2.33)
Procedural complications^†^	684/2942 (23%)	337/1565 (22%)	OR	1.22 (0.86 to 1.73)	1.17 (0.84 to 1.64)
Workflow outcomes					
First-pass reperfusion	604/3011 (20%)	264/1747 (15%)	OR	1.23 (0.92 to 1.63)	1.24 (0.92 to 1.68)
Duration procedure (min)– median (IQR)	47 (29–72), 3017	49 (31–73), 1646	β	0.80 (−5.09 to 6.68)	0.88 (−5.21 to 6.97)
Door to groin (min)– median (IQR)	56 (35–88), 2896	55 (28–78), 1658	β	2.73 (−12.0–17.5)	4.24 (−7.09 to 15.57)

EE: effect estimate; mRS: modified Rankin Scale; NIHSS: National Institutes of Health Stroke Scale; ICH: intracranial hemorrhage; ER: emergency room; ASPECTS: Alberta Stroke Program Early CT Score.

In case of missing values, the numbers in this table are noted as “no./total no.,” or “median (IQR), total no.”

§ The reported intervals were not corrected for multiple testing.

* Recanalization rates in 2018 were 73% for level 1 centers and 65% for level 2 centers.

† Scored on digital subtraction angiography.

Adjusted for: age; sex; NIHSS score at baseline; pre-stroke mRS score; diabetes mellitus; atrial fibrillation; hypertension; previous stoke; baseline blood glucose concentration; administration of intravenous thrombolysis; duration between onset to ER arrival at the intervention center; collateral grade; occlusion segment; and ASPECTS at baseline.

Our data did show an increased frequency of successful recanalization in level 1 centers, compared to level 2 centers in both the univariable analysis (OR 1.59, 95% CI: 1.11 to 2.27) and the multivariable analysis (aOR 1.60, 95% CI: 1.10 to 2.33) (Table 2). This difference was at least partially explained by CV because after adding this variable to the model, the effect was no longer significant (aOR 1.31, 95% CI: 0.95 to 1.81).

Furthermore, we did not observe any significant differences between center levels regarding NIHSS score at 24–48 h post EVT (aβ: 0.31, 95% CI: −0.52 to 1.14); mortality at 90 days (aOR: 1.13, 95% CI: 0.91 to 1.41); DTGT (aβ: 4.24, 95% CI: −7.09 to 15.57); duration of the procedure (aβ: 0.88, 95% CI: −5.21 to 6.97); the frequency of achieving first-pass reperfusion (aOR: 1.24, 95% CI: 0.92 to 1.68) or any of the safety outcomes (Table 2).

### Sensitivity analyses

The results of our primary analysis did not change after performing a sensitivity analysis in which we additionally adjusted for the EXPfreq of the interventionist (acOR 0.83, 95% CI: 0.65 to 1.06).

In our sensitivity analysis, the probability of recanalization was also higher in patients treated in level 1 centers (aOR 1.98, 95% CI: 1.31–2.99), and after additionally adjusting for CV this effect maintained significant (aOR 1.58, 95% CI: 1.14–2.20). After adjusting for both CV and EXPfreq of the interventionist this did not seem to influence the result (aOR 1.59, 95% CI: 1.14–2.20).

## Discussion

We observed no significant differences in functional outcomes at 90 days, early neurological outcomes, or safety outcomes between level 1 and level 2 centers performing EVT for AIS. We did observe a higher frequency of successful recanalization in level 1 centers compared to level 2 intervention centers. This association does not seem to be independent of the annual number of EVTs per center because the association was no longer significant after taking this variable into account. Our sensitivity analysis did not show an important modification of our model by EXPfreq of the interventionist.

We did not find any previous literature comparing the efficacy of level 1 and level 2 centers. There are, however, studies that have focused on the effect of EVT volume on outcomes. Using the MR CLEAN Registry data, we have previously shown that a higher CV was associated with better short-term neurological outcomes.^8^ Another study also found an association between a higher CV and lower mortality rates.^9^ One study compared high- and low-volume centers and demonstrated that patients treated in high-volume centers had higher recanalization rates and higher probabilities of good functional outcomes.^2,10^ On the whole, these studies imply a beneficial effect of centralization toward higher volume centers, regardless of center level.

It is important, though, to realize that centralization might lead to treatment delays, while the treatment benefit of EVT for AIS is very time-dependent.^11^ This was demonstrated by a Dutch study that showed that transferring EVT-eligible patients was associated with a treatment delay and worse functional outcomes compared to direct treatment in the intervention center.^3^ As between-center distances and travel times in the Netherlands are relatively short, these results underline the importance of the time factor in decisions regarding transfers.^3^ However, that study did not take center volumes into consideration.

Based on the aforementioned studies, the effects of centralization on outcomes after EVT for AIS seem to depend on the trade-off between the beneficial effects of high center volumes, and the negative effects of treatment delays associated with patient transfer. One study investigated this trade-off and indicated that the benefits of high annual EVT CV may outweigh the detrimental effects of delayed treatment.^12^

In our study population, we observed that patients treated in a level 1 center had on average fewer co-morbidities at baseline, compared to patients treated in a level 2 center. Since level 1 centers treat more referral patients compared to level 2 centers, some selection may have occurred in the referral of patients to these centers, which might explain the somewhat more favorable baseline characteristics. The larger number of transferred patients treated in the level 1 centers may also contribute to the delay in onset to ER arrival observed in level 1 centers.

### Strengths and limitations

An important strength of the current study is the large clinical dataset that represents daily clinical practice. In the Netherlands, referral patterns are not based on center levels and both level 1 and level 2 centers perform EVT for AIS since this treatment became the standard of care. Furthermore, both center types receive transferred patients only from nearby level 3 centers, thereby avoiding the bias that may otherwise have occurred.

Moreover, in our study, differences in CV are minimal. Although, the CV of level 1 centers was on average higher compared to level 2 centers, some level 2 centers treated more patients on an annual basis than some level 1 centers, which can be seen in Table 1 by the overlapping IQRs. Additionally, between-center travel times are relatively short.^13^ Our results thus show the comparison between level 1 and level 2 centers in a scenario in which differences in CV and treatment delays between centers are minimal. In addition, we corrected our models for both possible confounders. Therefore, the current situation was an optimal situation to compare level 1 and level 2 centers – with minimal confounding.

Nevertheless, certain limitations need to be addressed. We should be careful with inferences based on the reported intervals since we did not adjust for multiplicity. Moreover, results are especially generalizable to urban areas and might be less generalizable to centers in more rural environments and to situations in which centers achieve a much lower or higher CV. Furthermore, patients in the Netherlands are treated by both neuro- and non-neuro interventionists, irrespective of center level. This reduces possible confounding based on the specialty of the treating interventionist, but it may also hamper generalizability to other countries where level 1 and level 2 centers differ structurally with respect to which specialist performs the EVT procedures. However, the results of a recent study suggest that revascularization rates and functional outcomes do not differ significantly between patients that are treated by interventional radiologists and patients that are treated by neuro-interventional physicians.^14^

## Conclusion

In this observational study, we found no significant differences in 90-day functional outcomes, short-term neurological outcomes, or workflow outcomes between AIS patients treated with EVT in level 1 and level 2 centers. Recanalization rates did not significantly differ between center types after additional adjustments were made for center volume. Therefore, our data showed no support for the notion that EVT treatment in level 1 centers should be preferred over treatment in level 2 centers as long as center volume as a predictor is respected. Based on our findings and previous literature, we thus recommend future guidelines and recommendations regarding referral patterns to focus on the trade-off between center volume and between-center travel times and not the center level.

## Supplemental Material

sj-docx-1-eso-10.1177_23969873221145771 – Supplemental material for Association between type of intervention center and outcomes after endovascular treatment for acute ischemic stroke: Results from the MR CLEAN Registry Click here for additional data file. Supplemental material, sj-docx-1-eso-10.1177_23969873221145771 for Association between type of intervention center and outcomes after endovascular treatment for acute ischemic stroke: Results from the MR CLEAN Registry by Susanne GH Olthuis, Wouter H Hinsenveld, Florentina ME Pinckaers, Marzyeh Amini, Hester F Lingsma, Julie Staals, Tobien HCML Schreuder, Wouter J Schonewille, Lonneke SF Yo, Yvo BWEM Roos, Alida A Postma, Diederik WJ Dippel, Wim H van Zwam, Robert J van Oostenbrugge and Inger R de Ridder in European Stroke Journal
