# Supplementary material for: Association between type of intervention center and outcomes after endovascular treatment for acute ischemic stroke: Results from the MR CLEAN Registry
Source: Eur Stroke J. 2022 Dec 22;8(1):224–30. doi: 10.1177/23969873221145771 (PMC10069206; doi:10.1177/23969873221145771)
Supplement: sj-docx-1-eso-10.1177_23969873221145771 – Supplemental material for Association between type of intervention center and outcomes after endovascular treatment for acute ischemic stroke: Results from the MR CLEAN Registry [file sj-docx-1-eso-10.1177_23969873221145771.docx]

**SUPPLEMENTAL MATERIAL**

**Table of contents**

- Figure S1. Histogram of yearly center volumes - per center type.
- List of group authors


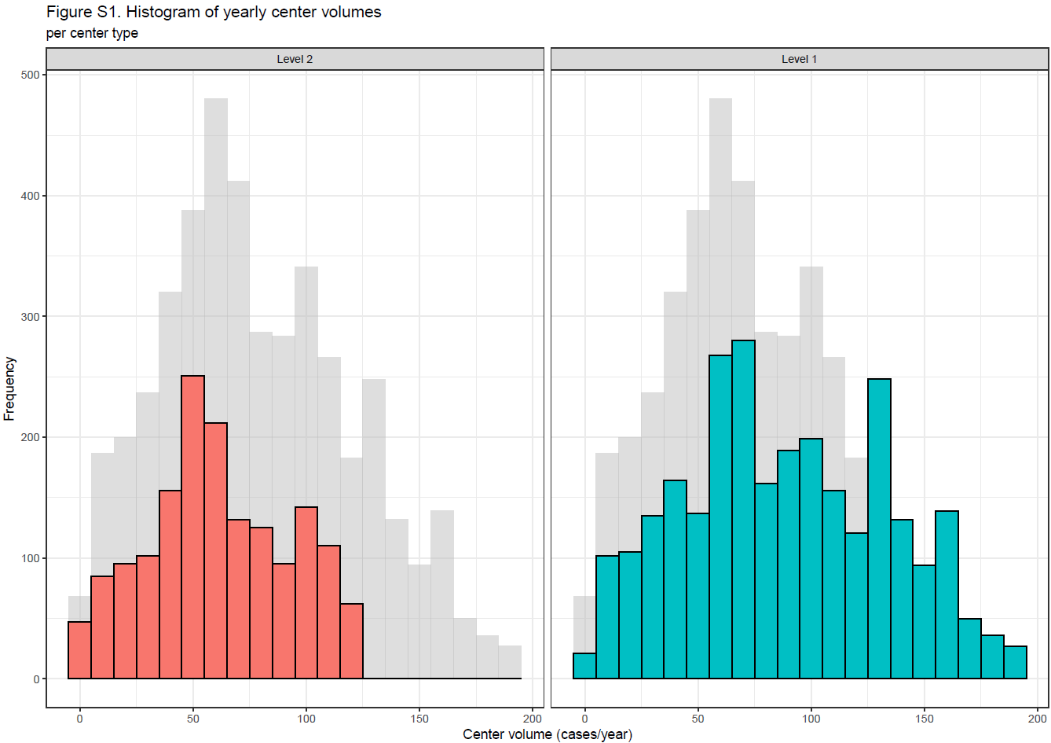


**Figure S1. Histogram of yearly center volumes- per center type**

Center volume was defined as the number of patients treated with EVT in the center in the previous year (calculated per current procedure/patient). Data on center volumes are shown for patients treated in level 2 (left panel) and level 1 (right panel) centers separately. The gray histograms - shown in both panels - represent the entire study population (from both level 1 and level 2 centers).

**List of group authors**

MR CLEAN (Multicenter Randomized Controlled Trial of Endovascular Treatment for Acute Ischemic Stroke in the Netherlands) Registry Investigators – group authors

Executive committee

Diederik W.J. Dippel^1^; Aad van der Lugt^2^; Charles B.L.M. Majoie^3^; Yvo B.W.E.M. Roos^4^; Robert J. van Oostenbrugge^5,44^; Wim H. van Zwam^6,44^; Jelis Boiten^14^; Jan Albert Vos^8^

Study coordinators

Ivo G.H. Jansen^3^; Maxim J.H.L. Mulder^1,2^; Robert- Jan B. Goldhoorn^5,6,44^; Kars C.J. Compagne^2^; Manon Kappelhof^3^; Josje Brouwer^4^; Sanne J. den Hartog^1,2,40^; Wouter H. Hinsenveld ^5,6^; Lotte van den Heuvel^1,40^.

Local principal investigators

Diederik W.J. Dippel^1^; Bob Roozenbeek^1^; Aad van der Lugt^2^; Pieter Jan van Doormaal^2^, Charles B.L.M. Majoie^3^; Yvo B.W.E.M. Roos^4^; Bart J. Emmer^3^; Jonathan M. Coutinho^4^; Wouter J. Schonewille^7^; Jan Albert Vos^8^; Marieke J.H. Wermer^9^; Marianne A.A. van Walderveen^10^; Adriaan C.G.M. van Es^10^; Julie Staals^5,44^; Robert J. van Oostenbrugge^5,44^; Wim H. van Zwam^6,44^; Jeannette Hofmeijer^11^; Jasper M. Martens^12^; Geert J. Lycklama à Nijeholt^13^; Jelis Boiten^14^; Sebastiaan F. de Bruijn^15^; Lukas C. van Dijk^16^; H. Bart van der Worp^17^; Rob H. Lo^18^; Ewoud J. van Dijk^19^; Hieronymus D. Boogaarts^20^; J. de Vries^22^; Paul L.M. de Kort^21^; Julia van Tuijl^21^; Issam Boukrab^26^; Jo P. Peluso^26^; Puck Fransen^22^; Jan S.P. van den Berg^22^; Heleen M. den Hertog^22^; Boudewijn A.A.M. van Hasselt^23^; Leo A.M. Aerden^24^; René J. Dallinga^25^; Maarten Uyttenboogaart^28^; Omid Eschgi^29^; Reinoud P.H. Bokkers^29^; Tobien H.C.M.L. Schreuder^30^; Roel J.J. Heijboer^31^; Koos Keizer^32^; Rob A.R. Gons^32^; Lonneke S.F. Yo^33^; Emiel J.C. Sturm^35,47^, Tomas Bulut^35^; Paul J.A.M. Brouwers^34^; Anouk D. Rozeman^42^; Otto Elgersma^41^, Michel J.M. Remmers^43^; Thijs E.A.M. de Jong^46^.

Imaging assessment committee

Charles B.L.M. Majoie^3^(chair); Wim H. van Zwam^6,44^; Aad van der Lugt^2^; Geert J. Lycklama à Nijeholt^13^; Marianne A.A. van Walderveen^10^; Marieke E.S. Sprengers^3^; Sjoerd F.M. Jenniskens^27^; René van den Berg^3^; Albert J. Yoo^38^; Ludo F.M. Beenen^3^; Alida A. Postma^6.45^; Stefan D. Roosendaal^3^; Bas F.W. van der Kallen^13^; Ido R. van den Wijngaard^13^; Adriaan C.G.M. van Es^10^; Bart J. Emmer^,3^; Jasper M. Martens^12^; Lonneke S.F. Yo^33^; Jan Albert Vos^8^; Joost Bot^36^; Pieter-Jan van Doormaal^2^; Anton Meijer^27^; Elyas Ghariq^13^; Reinoud P.H. Bokkers^29^; Marc P. van Proosdij^37^; G. Menno Krietemeijer^33^; Jo P. Peluso^26^; Hieronymus D. Boogaarts^20^; Rob Lo^18^;Wouter Dinkelaar^41^; Auke P.A. Appelman^29^; Bas Hammer^16^; Sjoert Pegge^27^; Anouk van der Hoorn^29^; Saman Vinke^20^; Sandra Cornelissen^2^; Christiaan van der Leij^6^; Rutger Brans^6^; Jeanette Bakker^41^; Maarten Uyttenboogaart^28^; Miou Koopman^3^; Lucas Smagge^2^; Olvert A. Berkhemer^1,3,6^; Jeroen Markenstein^3^; Eef Hendriks^3^; Patrick Brouwer^10^; Dick Gerrits^35^.

Writing committee

Diederik W.J. Dippel^1^(chair); Aad van der Lugt^2^; Charles B.L.M. Majoie^3^; Yvo B.W.E.M. Roos^4^; Robert J. van Oostenbrugge^5,44^; Wim H. van Zwam^6,44^; Geert J. Lycklama à Nijeholt^13^; Jelis Boiten^14^; Jan Albert Vos^8^; Wouter J. Schonewille^7^; Jeannette Hofmeijer^11^; Jasper M. Martens^12^; H. Bart van der Worp^17^; Rob H. Lo^18^

Adverse event committee

Robert J. van Oostenbrugge^5,44^(chair); Jeannette Hofmeijer^11^; H. Zwenneke Flach^23^

Trial methodologist

Hester F. Lingsma^40^

Research nurses / local trial coordinators

Naziha el Ghannouti^1^; Martin Sterrenberg^1^; Wilma Pellikaan^7^; Rita Sprengers^4^; Marjan Elfrink^11^; Michelle Simons^11^; Marjolein Vossers^12^; Joke de Meris^14^; Tamara Vermeulen^14^; Annet Geerlings^19^; Gina van Vemde^22^; Tiny Simons^30^; Gert Messchendorp^28^; Nynke Nicolaij^28^; Hester Bongenaar^32^; Karin Bodde^24^; Sandra Kleijn^34^; Jasmijn Lodico^34^; Hanneke Droste^34^; Maureen Wollaert^5^; Sabrina Verheesen^5^; D. Jeurrissen^5^; Erna Bos^9^; Yvonne Drabbe^15^; Michelle Sandiman^15^; Nicoline Aaldering^11^; Berber Zweedijk^17^; Jocova Vervoort^21^; Eva Ponjee^22^; Sharon Romviel^19^; Karin Kanselaar^19^; Denn Barning^10^ ; Laurine van der Steen^3^.

Clinical/imaging data aquisition

Esmee Venema^40^; Vicky Chalos^1,40^; Ralph R. Geuskens^3^; Tim van Straaten^19^; Saliha Ergezen^1^; Roger R.M. Harmsma^1^; Daan Muijres^1^; Anouk de Jong^1^; Olvert A. Berkhemer^1,3,6^; Anna M.M. Boers^3,39^; J. Huguet^3^; P.F.C. Groot^3^; Marieke A. Mens^3^; Katinka R. van Kranendonk^3^; Kilian M. Treurniet^3^; Manon L. Tolhuisen^3,39^; Heitor Alves^3^; Annick J. Weterings^3^; Eleonora L.F. Kirkels^3^; Eva J.H.F. Voogd^11^; Lieve M. Schupp^3^; Sabine L. Collette^28,29^; Adrien E.D. Groot^4^; Natalie E. LeCouffe^4^; Praneeta R. Konduri^39^; Haryadi Prasetya^39^; Nerea Arrarte-Terreros^39^; Lucas A. Ramos^39^ ; Nikki Boodt^1,2,40^; Anne F.A.V Pirson^5^; Agnetha A.E. Bruggeman^3^; Nadinda A.M. van der Ende ^1,2^, Rabia Deniz^3^, Susanne G.H. Olthuis^5,44^, Floor Pinckaers^6,44^

List of affiliations

Department of Neurology^1^, Radiology^2^, Public Health^40^, Erasmus MC University Medical Center;

Department of Radiology and Nuclear Medicine^3^, Neurology^4^, Biomedical Engineering & Physics^39^, Amsterdam UMC, location University of Amsterdam;

Department of Neurology^5^, Radiology & Nuclear Medicine^6^, Maastricht University Medical Center+; School for Cardiovascular Diseases Maastricht (CARIM)^44^_;_ and MHeNs School for Mental Health and Neuroscience, Maastricht, the Netherlands^45^;

Department of Neurology^7^, Radiology^8^, Sint Antonius Hospital, Nieuwegein;

Department of Neurology^9^, Radiology^10^, Leiden University Medical Center;

Department of Neurology^11^, Radiology^12^, Rijnstate Hospital, Arnhem;

Department of Radiology^13^, Neurology^14^, Haaglanden MC, the Hague;

Department of Neurology^15^, Radiology^16^, HAGA Hospital, the Hague;

Department of Neurology^17^, Radiology^18^, University Medical Center Utrecht;

Department of Neurology^19^, Neurosurgery^20^, Radiology^27^, Radboud University Medical Center, Nijmegen;

Department of Neurology^21^, Radiology^26^, Elisabeth-TweeSteden ziekenhuis, Tilburg;

Department of Neurology^22^, Radiology^23^, Isala Klinieken, Zwolle;

Department of Neurology^24^, Radiology^25^, Reinier de Graaf Gasthuis, Delft;

Department of Neurology^28^, Radiology^29^, University Medical Center Groningen;

Department of Neurology^30^, Radiology^31^, Zuyderland Medical Center, Heerlen;

Department of Neurology^32^, Radiology^33^, Catharina Hospital, Eindhoven;

Department of Neurology^34^, Radiology^35^, Medisch Spectrum Twente, Enschede, (currently Deventer Hospital^47^);

Department of Radiology^36^, Amsterdam UMC, Vrije Universiteit van Amsterdam, Amsterdam;

Department of Radiology^37^, Noordwest Ziekenhuisgroep, Alkmaar;

Department of Radiology^38^, Texas Stroke Institute, Texas, United States of America;

Department of Neurology^42^, Radiology^41^, Albert Schweitzer Hospital, Dordrecht;

Department of Neurology^43^, Radiology^46^, Amphia Hospital, Breda.
